# Using Nudges to Reduce Missed Appointments in Primary Care and Mental Health: a Pragmatic Trial

**DOI:** 10.1007/s11606-023-08131-5

**Published:** 2023-06-20

**Authors:** Alan R. Teo, Meike Niederhausen, Robert Handley, Emily E. Metcalf, Aaron A. Call, R. Lorie Jacob, Brian J. Zikmund-Fisher, Steven K. Dobscha, Peter J. Kaboli

**Affiliations:** 1 VA Portland Health Care System, HSR&D Center to Improve Veteran Involvement in Care, 3710 SW US Veterans Hospital Road (R&D 66), Portland, OR USA; 2 Department of Psychiatry, Oregon Health & Science University, Portland, OR USA; 3 Oregon Health & Science University - Portland State University (OHSU-PSU) School of Public Health, Oregon Health & Science University, Portland, OR USA; 4 Department of Health Behavior of Health Education, School of Public Health, University of Michigan, Ann Arbor, MI USA; 5 Department of Internal Medicine, School of Medicine, University of Michigan, Ann Arbor, MI USA; 6 Center for Bioethics and Social Sciences in Medicine, University of Michigan, Ann Arbor, MI USA; 7 Comprehensive Access and Delivery Research and Evaluation Center, Iowa City Veterans Affairs Healthcare System, Iowa City, IA USA; 8 Division of General Internal Medicine, Department of Internal Medicine, University of Iowa Carver College of Medicine, Iowa City, IA USA

**Keywords:** no-show, behavioral economics, adherence, treatment engagement

## Abstract

**Background:**

Missed appointments (“no-shows”) are a persistent and costly problem in healthcare. Appointment reminders are widely used but usually do not include messages specifically designed to nudge patients to attend appointments.

**Objective:**

To determine the effect of incorporating nudges into appointment reminder letters on measures of appointment attendance.

**Design:**

Cluster randomized controlled pragmatic trial.

**Patients:**

There were 27,540 patients with 49,598 primary care appointments, and 9420 patients with 38,945 mental health appointments, between October 15, 2020, and October 14, 2021, at one VA medical center and its satellite clinics that were eligible for analysis.

**Interventions:**

Primary care (*n* = 231) and mental health (*n* = 215) providers were randomized to one of five study arms (four nudge arms and usual care as a control) using equal allocation. The nudge arms included varying combinations of brief messages developed with veteran input and based on concepts in behavioral science, including social norms, specific behavioral instructions, and consequences of missing appointments.

**Main Measures:**

Primary and secondary outcomes were missed appointments and canceled appointments, respectively.

**Statistical Analysis:**

Results are based on logistic regression models adjusting for demographic and clinical characteristics, and clustering for clinics and patients.

**Key Results:**

Missed appointment rates in study arms ranged from 10.5 to 12.1% in primary care clinics and 18.0 to 21.9% in mental health clinics. There was no effect of nudges on missed appointment rate in primary care (OR = 1.14, 95%CI = 0.96–1.36, *p* = 0.15) or mental health (OR = 1.20, 95%CI = 0.90–1.60,* p* = 0.21) clinics, when comparing the nudge arms to the control arm. When comparing individual nudge arms, no differences in missed appointment rates nor cancellation rates were observed.

**Conclusions:**

Appointment reminder letters incorporating brief behavioral nudges were ineffective in improving appointment attendance in VA primary care or mental health clinics. More complex or intensive interventions may be necessary to significantly reduce missed appointments below their current rates.

**Trial Number:**

ClinicalTrials.gov, Trial number NCT03850431.

**Supplementary Information:**

The online version contains supplementary material available at 10.1007/s11606-023-08131-5.

## BACKGROUND

Missed appointments, frequently referred to as “no-shows,” are a longstanding challenge to providers and administrators in every healthcare system. In the Department of Veterans Affairs (VA), the largest integrated healthcare system in the USA, the national missed appointment rate was 14.9% from October 2020 to September 2021, resulting in 10,109,148 missed appointments.^1^ Patients prone to miss appointments are a vulnerable population, known to have higher rates of preventable hospitalizations, emergency department visits, and all-cause mortality.^2,3^

Like many behaviors, the potential reasons behind a missed appointment are myriad, but broadly speaking they can include patient-related characteristics (e.g., history of psychiatric disorders^4^), features of the healthcare system (e.g., scheduling systems^5^), and factors that lie in between the two (e.g., how long prior to the scheduled date the appointment was scheduled).^6^ Nonetheless, simply forgetting the appointment is perhaps the single most common reason for a missed appointment.^7^ Given this, it is not surprising that appointment reminders are employed more or less universally across healthcare systems—a straightforward, first line of defense against missed appointments. Traditionally, appointment reminders provide a “bare bones” message about the time, date, and place of an appointment, which is more or less how they have been for at least the last 40 years.^8–10^

One of the core ideas of behavioral economics is the idea of supporting behavior change through the use of nudges: small alterations in how options are presented that are designed to influence a person’s decision making without restricting their choices.^11,12^ Several studies have found promising impacts on appointment attendance through application of nudges that leveraged principles such as people’s general desire to avoid physical or emotional losses (“loss aversion”) and adhere to prevalent social norms.^13–16^ Specifically, a randomized controlled trial in outpatient specialty clinics in the UK’s National Health Service found that specifying the cost of a missed appointment to the healthcare system significantly reduced the rate of missed appointments, and including the descriptive social norm that most patients attended their appointment significantly increased the rate of canceling appointments in advance, thus potentially averting missed appointments.^17^ An Israeli study found several messages effective at reducing missed appointments, the most effective being a text message appointment reminder designed to evoke emotional guilt (“Not showing up to your appointment without canceling in advance delays hospital treatment for those who need medical aid.^18^) Finally, in the USA, a randomized trial of 360 veterans at a single VA medical center who were referred to specialty mental health for depression found that patients who received an appointment reminder letter containing a bolded, gain-framed message (“If you go to your appointment, you will learn ways to improve your mood and emotional well-being. Also, if you see your provider, he/she will be able to work with you to help you get the most out of your treatment.”) were more likely to attend their appointments, compared to those who received a routine letter without the additional message.^19^

To date, there have been no large-scale trials of behavioral economics-informed appointment reminders in the USA. Moreover, the effect of other types of nudges, or combining nudges, is not known. The aim of this study was to conduct a large-scale pragmatic trial within the VA to evaluate the effect of a series of nudges on missed and canceled appointments. We hypothesized that the intervention, as compared with usual care, would have a lower missed appointment rate and higher canceled appointment rate. We also hypothesized that effect size of nudges would vary by intervention arm such that some types of nudges would have a larger proportional effect than others.

## METHODS

### Design and Setting

We conducted a cluster randomized controlled pragmatic trial at the VA Portland Health Care System, which cares for approximately 85,000 patients across Oregon and southwest Washington, including two medical centers (Portland and Vancouver) and six satellite clinics (Hillsboro, Fairview, West Linn, Salem, Bend, and North Coast).

Pragmatic trial features included broad eligibility criteria, intervention implementation integrated with usual care, usual care as the comparison condition, and outcome assessment using electronic health record data.^20,21^ The trial began December 2019 but was halted in March 2020 due to the COVID-19 pandemic. It resumed October 2020 and lasted 1 year until October 2021. Due to cohort effects and a small sample size, pre-COVID trial data were not included. The VA Portland Healthcare System Institutional Review Board and Research and Development Service approved the study, which was registered with ClinicalTrials.gov (Trial number NCT03850431) prior to initiation.

For inclusion, outpatient appointments had to be as follows: (1) in either primary care or mental health; and (2) scheduled at least 2 weeks before the appointment date (the minimum length of time used to determine whether an appointment reminder is mailed). We used “stop codes,” 3-digit identifiers used within VA,^22^ to identify appointments as primary care (codes: 322, 323, and 338) or mental health (codes: 502, 513, 527, 534, 542, 545, and 562). Patients were included if they had at least one appointment meeting the above criteria.

### Randomization and Masking

We conducted randomization at the provider (not patient) level by allocating to one of four nudge (intervention) arms or control arm (1:1:1:1:1), and stratifying by setting (primary care or mental health) and location (Portland, Vancouver, or other). A statistician (MN) masked to intervention information used block randomization with random block sizes of 5, 10, or 15, assigning providers to one of five study arms using the blockrand (v 1.5) R package^23^.

### Interventions

**Figure 1:**
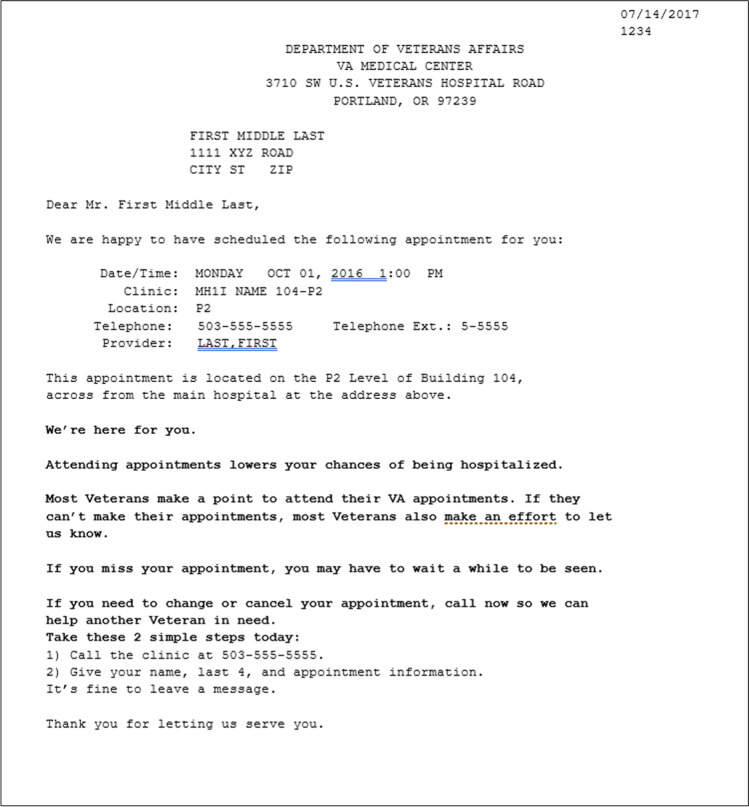
Example intervention appointment letter (arm D)

We developed four nudge arms using a combination of theory, empirical data, and veteran input. We began with candidate messages organized around broad concepts in behavioral science that seemed plausible motivators for a patient to attend and/or cancel their appointment: social norms, behavioral instructions and intentions, consequences for self, and consequences for others (Table 1). We then employed a user-centered design (UCD) process as a strategy to make our interventions patient-centered and minimize potential risks and unintended consequences as reported previously.^24^ During the UCD process, we conducted iterative waves of interviews with veterans, eliciting feedback on nudge language and the draft interventions. Interviews influenced key aspects of intervention content, ultimately resulting in removal or revision of several candidate nudge messages, generation of new messages, and selection of specific combinations of messages for each intervention. The four finalized interventions are summarized in Table 1. To illustrate how the intervention appeared in practice, Fig. 1 presents the letter for arm D, which contained a combination of all the nudges, as seen by a veteran with a primary care appointment.

**Table 1:** Description of Study Arms

Short name for study arm	Principle	Message
Usual Care (Arm B)	Usual Care	Not applicable
Consequences for Self (Arm A)	Caring^48–50^	**We’re here for you**
	Consequences for Self^13,14^	**If you miss your appointment, you may have to wait a while to be seen** Attending appointments lowers your chances of being hospitalized
	Behavioral Instructions and Intentions^51,52^	**Need to change or cancel your appointment?** **Take these 2 simple steps today:** 1) Call the clinic at 503–555-5555 2) Give your name, last 4, and appointment information. It’s fine to leave a message
Consequences for Others (Arm C)	Caring^48–50^	1) **We’re here for you**
	Behavioral Instructions and Intentions^51,52^ + Consequences for Others^53,54^	**If you need to change or cancel your appointment, call now so we can help another Veteran in need** **Take these 2 simple steps today:** 1) Call the clinic at 503–555-5555 2) Give your name, last 4, and appointment information. It’s fine to leave a message.**”**
Social Norms (Arm E)	Social Norms^15,16^	**Most Veterans make a point to attend their VA appointments. If they can’t make their appointments, most Veterans also make an effort to let us know**
	Behavioral Instructions and Intentions^51,52^	**Need to change or cancel your appointment?** **Take these 2 simple steps today:** 1) Call the clinic at 503–555-5555 2) Give your name, last 4, and appointment information. It’s fine to leave a message
Combination of All Nudges (Arm D)	Caring^48–50^	**We’re here for you**
	Social Norms^15,16^	**Most Veterans make a point to attend their VA appointments. If they can’t make their appointment, most Veterans also make an effort to let us know**
	Behavioral Instructions and Intentions^51,52^ + Consequences for Others^53,54^	**If you need to change or cancel your appointment, call now so we can help another Veteran in need** **Take these 2 simple steps today:** 1) Call the clinic at 503–555-5555 2) Give your name, last 4, and appointment information. It’s fine to leave a message
	Consequences for Self^13,14^	**If you miss your appointment, you may have to wait a while to be seen** Attending appointments lowers your chances of being hospitalized

Consistent with our pragmatic trial approach, interventions were delivered as they would be in routine clinical practice. This meant scheduling staff, who were unaware of study hypotheses or procedures, were responsible for preparation and mailing of templated appointment reminders. For mental health clinics, routine practice included not only an appointment reminder but also a letter after a missed appointment; therefore, nudge messages for each intervention were incorporated into these letters too. One unmasked research team member (EM) worked with clinic staff to modify, maintain, and audit letter templates.

### Control Arm

The control arm consisted of routine appointment reminder letters providing the clinic name, location, provider, date and time of appointment, and contact information. Since reminder *letters* were the focus of this trial, patients in all study arms continued to receive automated phone (audio), text, and postcard reminders (primary care only) without the nudge messages.

### Main Measures

We used the VA Corporate Data Warehouse to identify eligible appointments and coded as completed, canceled (by patient), or missed (“no-show”). Appointments that could not be classified due to incomplete data were excluded (0.05%). The *primary outcome* was whether a patient missed an appointment. In accordance with VA policy, an appointment was considered missed if marked by clinic staff as a “no-show” or if canceled by the patient or clinic after the appointment time. The missed appointment rate equals the number of missed appointments divided by the number of completed and missed appointments. Our *secondary outcome* was whether a patient canceled their appointment. As clinics sometimes cancel appointments for administrative reasons and the focus of our trial was patient behavior, we examined only appointments canceled by patients with a timestamp prior to the scheduled appointment time and calculated a cancelation rate accordingly. The canceled appointment rate equals the number of canceled appointments divided by the number of completed, canceled, and missed appointments.

### Statistical Analyses

We first compared patient demographics and clinical characteristics between the control and intervention groups (both combined and individually), using counts and percentages for categorical variables and means and standard deviations (SD) for quantitative variables. We used standardized mean differences (SMD)^25^ to compare the effect size between the control and combined intervention groups. For patients with multiple appointments during the study period, we used data from their first appointment in primary care and mental health, respectively, to summarize baseline patient-level characteristics.

The primary analyses compared the odds of a missed appointment between the control and the combined intervention groups using appointment-level logistic regression models with non-nested clustering for repeated appointments among providers and patients^26^. Primary care and mental health appointments were analyzed separately. We ran unadjusted and adjusted models, which included age, gender (missing values for self-identified gender were imputed with sex), race (collapsed as white, non-white, and unknown), ethnicity, rurality, VA disability rating,^27^ mental health diagnosis in the prior 2 years (i.e., depression, PTSD, substance use disorder), Elixhauser comorbidity score,^28^ Care Assessment Need (CAN) score^29^ (estimated probability of readmission or death within 90 days in primary care sample only), provider type, appointment age, appointment modality (virtual vs. in-person), appointment location (Portland, Vancouver, or other 6 sites combined), and the number of prior visits for the patient at the provider’s clinic location in the past 2 years. We selected covariates based on significance in our models and prior literature demonstrating associations with missed appointments (e.g., demographic characteristics, rurality, disability rating as a proxy for socioeconomic and health insurance status, mental health history, appointment age).^4,30–32^ Variance inflation factors were checked for multicollinearity, and all were less than 2.15. Patient characteristics were non-varying, using the values from the first appointment if they had repeated appointments in either setting. We also ran separate models with intervention × gender and intervention × modality interactions. In addition to odds ratios, we calculated mean predicted probabilities and 95% confidence intervals for each intervention via 10,000 Monte Carlo simulations, using reference levels for categorical variables and mean values for continuous covariates.

We conducted two sensitivity analyses. To address the possibility of diminished effect after repeated exposure to the intervention, the first sensitivity analysis was restricted to each patient’s first appointment (separately for primary care and mental health). To provide a more accurate estimate of the standard errors, we used logistic generalized estimating equation models with clustering for providers using an exchangeable covariance structure.^33,34^ The second sensitivity analysis was restricted to primary care providers (in primary care) and therapists and prescribers (in mental health); this was done to avoid including appointments with other providers (e.g., nurses, pharmacists) who frequently have “group appointments” that did not receive appointment reminder letters. All analyses were repeated using all five study arms with the control group as the reference level, and again for the cancelation outcome. Analyses were run in R (v. 4.1.2)^35^ using the smd (v. 0.6.6),^36^ lmtest (v. 0.9–40),^37^ sandwich (v. 3.0.2)^26,38^ (vcovCL for two-way clustering), and geepack (v. 1.3.9)^39–41^ packages. All tests were two-sided with a significance threshold of 5%.

## RESULTS

### Patients and Clinics

**Figure 2:**
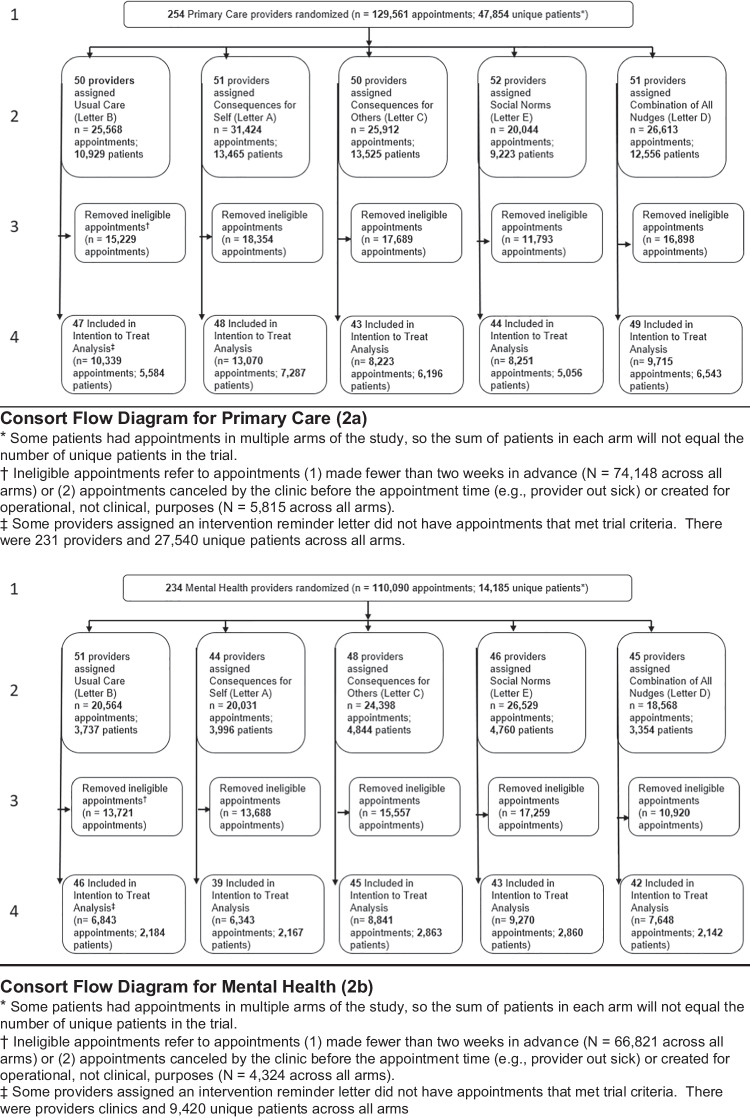
Consort flow diagrams for primary care (**a**) and mental health (**b**). **Consort Flow Diagram for Primary Care (2a)**. * Some patients had appointments in multiple arms of the study, so the sum of patients in each arm will not equal the number of unique patients in the trial. † Ineligible appointments refer to appointments (1) made fewer than two weeks in advance (N = 74,148 across all arms) or (2) appointments canceled by the clinic before the appointment time (e.g., provider out sick) or created for operational, not clinical, purposes (N = 5,815 across all arms). ‡ Some providers assigned an intervention reminder letter did not have appointments that met trial criteria. There were 231 providers and 27,540 unique patients across all arms. **Consort Flow Diagram for Mental Health (2b)**. * Some patients had appointments in multiple arms of the study, so the sum of patients in each arm will not equal the number of unique patients in the trial. † Ineligible appointments refer to appointments (1) made fewer than two weeks in advance (N = 66,821 across all arms) or (2) appointments canceled by the clinic before the appointment time (e.g., provider out sick) or created for operational, not clinical, purposes (N = 4,324 across all arms). ‡ Some providers assigned an intervention reminder letter did not have appointments that met trial criteria. There were providers clinics and 9,420 unique patients across all arms

Two hundred thirty-one primary care providers had 27,540 unique patients with a total of 49,598 appointments in the trial. Likewise, 215 mental health providers had 9420 unique patients with a total of 38,945 appointments. Thirty-six percent of primary care (70% of mental health) patients had more than one appointment included in the study. Of these, 13% (29%) had appointments with multiple providers. Because study arm randomization was at the provider level, this led to 11% (25%) of patients having appointments in more than one study arm within primary care and/or mental health. Consort flowcharts are presented in Fig. 2.

As shown in Tables 2 and 3, most patients were white, male, and not Hispanic or Latino, and resided in urban areas; mean age was 63.7 years (primary care) and 50.0 (mental health).

**Table 2:** Baseline Characteristics at the Patient Level for Primary Care

	Control vs. all nudge arms combined				Individual nudge arms			
	Overall	Control	Treatment	SMD^^^	Consequences for self	Consequences for others	Social norms	Combination of all nudges
Measure	*N* = 27,540*	*N* = 4916*	*N* = 22,624*		*N* = 6592*	*N* = 5759*	*N* = 4353*	*N* = 5920*
Age	63.7 (15.5)	65.6 (14.4)	63.3 (15.7)	0.150	64.8 (14.8)	62.8 (15.9)	62.9 (16.1)	62.6 (16.1)
Gender^†^				0.129				
Female	2783 (10.1%)	349 (7.1%)	2434 (10.8%)		564 (8.6%)	839 (14.6%)	444 (10.2%)	587 (9.9%)
Race				0.038				
White	22,016 (79.9%)	3888 (79.1%)	18,128 (80.1%)		5340 (81.0%)	4540 (78.8%)	3488 (80.1%)	4760 (80.4%)
American Indian or Alaska Native	357 (1.3%)	65 (1.3%)	292 (1.3%)		91 (1.4%)	71 (1.2%)	58 (1.3%)	72 (1.2%)
Asian	281 (1.0%)	49 (1.0%)	232 (1.0%)		60 (0.9%)	45 (0.8%)	70 (1.6%)	57 (1.0%)
Native Hawaiian or Pacific Islander	230 (0.8%)	37 (0.8%)	193 (0.9%)		46 (0.7%)	39 (0.7%)	45 (1.0%)	63 (1.1%)
Black or African American	891 (3.2%)	155 (3.2%)	736 (3.3%)		218 (3.3%)	172 (3.0%)	147 (3.4%)	199 (3.4%)
More than one race	307 (1.1%)	61 (1.2%)	246 (1.1%)		70 (1.1%)	59 (1.0%)	54 (1.2%)	63 (1.1%)
Unknown	3458 (12.6%)	661 (13.4%)	2797 (12.4%)		767 (11.6%)	833 (14.5%)	491 (11.3%)	706 (11.9%)
Ethnicity				0.046				
Hispanic or Latino	830 (3.0%)	118 (2.4%)	712 (3.1%)		183 (2.8%)	197 (3.4%)	138 (3.2%)	194 (3.3%)
Not Hispanic or Latino	23,285 (84.5%)	4188 (85.2%)	19,097 (84.4%)		5664 (85.9%)	4748 (82.4%)	3715 (85.3%)	4970 (84.0%)
Unknown	3425 (12.4%)	610 (12.4%)	2815 (12.4%)		745 (11.3%)	814 (14.1%)	500 (11.5%)	756 (12.8%)
Rurality				0.015				
Rural	9210 (33.4%)	1672 (34.0%)	7538 (33.3%)		2208 (33.5%)	2208 (38.3%)	1295 (29.7%)	1827 (30.9%)
Missing	1	0	1		0	0	0	1
VA service connection				0.040				
At least 50%	11,317 (41.1%)	1941 (39.5%)	9376 (41.4%)		2666 (40.4%)	2403 (41.7%)	1835 (42.2%)	2472 (41.8%)
Depression diagnosis in the prior two years				0.004				
Yes	6519 (23.7%)	1156 (23.5%)	5363 (23.7%)		1578 (23.9%)	1357 (23.6%)	1025 (23.5%)	1403 (23.7%)
PTSD diagnosis in the prior two years				0.034				
Yes	5947 (21.6%)	1005 (20.4%)	4942 (21.8%)		1422 (21.6%)	1339 (23.3%)	920 (21.1%)	1261 (21.3%)
Substance use disorder diagnosis in the prior two years				0.017				
Yes	3103 (11.3%)	532 (10.8%)	2571 (11.4%)		674 (10.2%)	661 (11.5%)	533 (12.2%)	703 (11.9%)
Elixhauser comorbidity score	14.0 (17.2)	15.4 (18.2)	13.7 (17.0)	0.102	14.7 (17.2)	12.1 (15.4)	13.9 (17.8)	13.8 (17.4)
CAN 90 score^‡^				0.048				
Lower risk	25,426 (93.0%)	4493 (91.9%)	20,933 (93.2%)		6084 (92.8%)	5418 (95.0%)	3987 (92.1%)	5444 (92.7%)
High risk	1925 (7.0%)	394 (8.1%)	1531 (6.8%)		471 (7.2%)	288 (5.0%)	344 (7.9%)	428 (7.3%)
Missing	189	29	160		37	53	22	48
Prior visits^§^	2.6 (3.4)	3.0 (4.4)	2.5 (3.1)	0.130	2.6 (3.5)	2.3 (2.4)	2.8 (3.4)	2.5 (3.0)

^*SMD*, standardized mean differences

^*^Mean (SD); *n* (%)

^†^Self-identified gender as indicated in the VA electronic health record, with missing values imputed with sex

^‡^Care Assessment Need score reflecting estimated probability of readmission or death at 90 days

^§^Number of prior visits for the patient at the provider’s clinic location in the past 2 years

**Table 3:** Baseline Characteristics at the Patient Level for Mental Health

	Control vs. interventions combined				Individual intervention arms			
	Overall	Control	Treatment	SMD^^^	Consequences for self	Consequences for others	Social norms	Combination of all nudges
Measure	*N* = 9420*	*N* = 1641*	*N* = 7779*		*N* = 1666*	*N* = 2305*	*N* = 2250*	*N* = 1558*
Age	50.0 (16.0)	50.1 (15.9)	50.0 (16.0)	0.005	49.5 (16.2)	51.4 (16.2)	49.4 (15.9)	49.3 (15.8)
Gender^†^				0.041				
Female	1848 (19.6%)	300 (18.3%)	1548 (19.9%)		403 (24.2%)	477 (20.7%)	362 (16.1%)	306 (19.6%)
Race				0.069				
White	7,240 (76.9%)	1,251 (76.2%)	5,989 (77.0%)		1,289 (77.4%)	1,823 (79.1%)	1,730 (76.9%)	1,147 (73.6%)
American Indian or Alaska Native	147 (1.6%)	19 (1.2%)	128 (1.6%)		25 (1.5%)	37 (1.6%)	37 (1.6%)	29 (1.9%)
Asian	140 (1.5%)	27 (1.6%)	113 (1.5%)		21 (1.3%)	33 (1.4%)	34 (1.5%)	25 (1.6%)
Native Hawaiian or Pacific Islander	98 (1.0%)	12 (0.7%)	86 (1.1%)		12 (0.7%)	29 (1.3%)	25 (1.1%)	20 (1.3%)
Black or African American	436 (4.6%)	83 (5.1%)	353 (4.5%)		69 (4.1%)	96 (4.2%)	101 (4.5%)	87 (5.6%)
More than one race	188 (2.0%)	33 (2.0%)	155 (2.0%)		30 (1.8%)	42 (1.8%)	47 (2.1%)	36 (2.3%)
Unknown	1171 (12.4%)	216 (13.2%)	955 (12.3%)		220 (13.2%)	245 (10.6%)	276 (12.3%)	214 (13.7%)
Ethnicity				0.020				
Hispanic or Latino	458 (4.9%)	78 (4.8%)	380 (4.9%)		86 (5.2%)	107 (4.6%)	99 (4.4%)	88 (5.6%)
Not Hispanic or Latino	7766 (82.4%)	1346 (82.0%)	6420 (82.5%)		1351 (81.1%)	1970 (85.5%)	1846 (82.0%)	1253 (80.4%)
Unknown	1196 (12.7%)	217 (13.2%)	979 (12.6%)		229 (13.7%)	228 (9.9%)	305 (13.6%)	217 (13.9%)
Rurality				0.101				
Rural	2473 (26.3%)	492 (30.0%)	1981 (25.5%)		443 (26.6%)	618 (26.8%)	581 (25.8%)	339 (21.8%)
Missing	1	1	0					
VA service connection				0.064				
At least 50%	5920 (62.8%)	1073 (65.4%)	4847 (62.3%)		1092 (65.5%)	1489 (64.6%)	1334 (59.3%)	932 (59.8%)
Depression diagnosis in the prior two years				0.049				
Yes	5663 (60.1%)	1019 (62.1%)	4644 (59.7%)		1003 (60.2%)	1442 (62.6%)	1270 (56.4%)	929 (59.6%)
PTSD diagnosis in the prior two years				0.071				
Yes	5221 (55.4%)	957 (58.3%)	4264 (54.8%)		902 (54.1%)	1327 (57.6%)	1210 (53.8%)	825 (53.0%)
Substance use disorder diagnosis in the prior two years				0.037				
Yes	2627 (27.9%)	480 (29.3%)	2147 (27.6%)		374 (22.4%)	601 (26.1%)	754 (33.5%)	418 (26.8%)
Elixhauser comorbidity score	14.7 (16.0)	15.5 (16.4)	14.5 (15.9)	0.057	14.1 (15.8)	15.2 (16.0)	14.9 (16.5)	13.5 (14.9)
Prior visits^‡^	5.5 (7.8)	5.4 (6.8)	5.5 (8.0)	− 0.012	6.0 (8.9)	5.3 (7.0)	5.0 (7.7)	5.9 (8.7)

^*SMD*, standardized mean differences

^*^Mean (SD); *n* (%)

^†^Self-identified gender as indicated in the VA electronic health record, with missing values imputed with gender

^‡^Number of prior visits for the patient at the provider’s clinic location in the past 2 years

### Primary Outcome

In both primary care and mental health, there was no significant effect of nudges on missed appointment rate (Table 4).

**Table 4:** Odds Ratios for Missed Appointments and Canceled Appointments in Primary Care and Mental Health

		Arm	N*	Count (percent)	Unadjusted model		Adjusted model^†^	
					OR (95% CI)	*p*-value	OR (95% CI)	*p*-value
Primary care	Missed appointment	All	46,111	5188 (11.3)				
		Control	9721	1159 (11.9)				
		All nudges	36,390	4029 (11.1)	0.91 (0.75, 1.10)	0.35	1.14 (0.96, 1.36)	0.15
		Consequences for self	12,321	1363 (11.1)	0.92 (0.73, 1.15)	0.44	1.11 (0.91, 1.35)	0.30
		Consequences for others	7492	788 (10.5)	0.86 (0.66, 1.12)	0.25	1.18 (0.95, 1.45)	0.13
		Combination of all nudges	8889	944 (10.6)	0.87 (0.67, 1.14)	0.31	1.12 (0.91, 1.37)	0.29
		Social norms	7688	934 (12.1)	1.01 (0.75, 1.37)	0.94	1.18 (0.92, 1.50)	0.18
	Canceled appointment	All	49,598	3487 (7.0)				
		Control	10,339	618 (6.0)				
		All nudges	39,259	2869 (7.3)	1.25 (0.83, 1.87)	0.29	0.92 (0.83, 1.03)	0.15
		Consequences for self	13,070	749 (5.7)	0.96 (0.58, 1.59)	0.88	0.91 (0.78, 1.07)	0.25
		Consequences for others	8223	731 (8.9)	1.55 (1.01, 2.37)	0.04	0.94 (0.81, 1.09)	0.41
		Combination of all nudges	9715	826 (8.5)	1.46 (0.97, 2.20)	0.07	0.95 (0.83, 1.08)	0.41
		Social norms	8251	563 (6.8)	1.16 (0.73, 1.86)	0.53	0.89 (0.77, 1.03)	0.11
Mental health	Missed appointment	All	35,420	6992 (19.7)				
		Control	6144	1104 (18.0)				
		All nudges	29,276	5888 (20.1)	1.15 (0.84, 1.58)	0.38	1.20 (0.90, 1.60)	0.21
		Consequences for Self	5750	1106 (19.2)	1.09 (0.74, 1.60)	0.67	1.14 (0.80, 1.64)	0.46
		Consequences for others	8131	1779 (21.9)	1.28 (0.91, 1.80)	0.16	1.33 (0.97, 1.82)	0.07
		Combination of all nudges	6984	1321 (18.9)	1.07 (0.76, 1.50)	0.71	1.17 (0.87, 1.58)	0.31
		Social norms	8411	1682 (20.0)	1.14 (0.82, 1.60)	0.44	1.15 (0.85, 1.57)	0.37
	Canceled appointment	All	38,945	3525 (9.1)				
		Control	6843	699 (10.2)				
		All nudges	32,102	2826 (8.8)	0.85 (0.65, 1.12)	0.24	0.83 (0.65, 1.06)	0.14
		Consequences for self	6343	593 (9.3)	0.91 (0.63, 1.30)	0.59	0.88 (0.66, 1.18)	0.40
		Consequences for others	8841	710 (8.0)	0.77 (0.53, 1.10)	0.15	0.74 (0.54, 1.03)	0.07
		Combination of all nudges	7648	664 (8.7)	0.84 (0.61, 1.14)	0.25	0.78 (0.60, 1.01)	0.06
		Social norms	9270	859 (9.3)	0.90 (0.65, 1.24)	0.51	0.93 (0.70, 1.25)	0.64

^*^N, number of appointments. For calculation of missed appointments, canceled appointments are not included

^†^Adjusted models include covariates for age, gender, race, ethnicity, rurality, VA service connection, depression diagnosis in prior 2 years, PTSD diagnosis in prior 2 years, substance use disorder diagnosis in prior 2 years, Elixhauser comorbidity score, CAN 90 score (PC models only), number of prior visits, type of provider, appointment age, appointment modality (in-person vs. virtual), and appointment location (Portland, Vancouver, other)

In primary care, the missed appointment rates for the intervention arms (all four nudge arms combined) and control group, respectively, were 11.1% and 11.9%. The odds ratio (OR) from the adjusted logistic regression model comparing the intervention arms to the control arm was 1.14 (95% CI = 0.96–1.36, *p* = 0.15; control arm: 10.9%, 95% CI = 10.0–13.7%; all nudge arms: 12.2%, 95% = 11.2–15.2%), which was the reverse relationship of the unadjusted percentages. When comparing nudge arms individually to the control arm, the OR’s ranged from 1.11 to 1.18 and were nonsignificant (control arm: 9.3%, 95% CI = 8.4–11.9%; nudge arms varied from 10.2 to 10.8%).

In mental health, the missed appointment rates for the intervention arms (all four nudge arms combined) and control group, respectively, were 20.1% and 18.0%. The OR from the adjusted logistic regression model comparing the intervention arms to the control arm was 1.20 (95%CI = 0.90–1.60, *p* = 0.21; control arm: 18.9%, 95% CI = 17.1–24.4%; all nudge arms: 21.8%, 95% = 20.4–25.9%). When comparing nudge arms individually to the control arm, the OR’s ranged from 1.14 to 1.33 and were nonsignificant (control arm: 19.2%, 95% CI = 17.3–24.9%; nudge arms varied from 21.4 to 23.9%).

### Secondary Outcome

In both primary care and mental health, there was no effect of nudges on canceled appointment rate (Table 4).

In primary care, the cancelation rates for the intervention arms (all four nudge arms combined) and control arm, respectively, were 7.3% and 6.0%, again with a “reversed” OR of 0.92 (95%CI = 0.83–1.03, *p* = 0.15) when comparing the intervention to the control in the adjusted logistic regression model. When comparing nudge arms individually to the control arm the OR’s ranged from 0.89 to 0.95, all nonsignificant.

In mental health, cancelation rates for the intervention arms (all four nudge arms combined) and control arm, respectively, were 8.8% and 10.2%, with an OR of 0.83 (95%CI = 0.65–1.06, *p* = 0.14) when comparing the intervention to the control group in the adjusted logistic regression model. When comparing nudge arms individually to the control arm the OR’s ranged from 0.74 to 0.93, all nonsignificant.

### Additional Analyses

In primary care, modality of appointment (OR = 0.74, 95%CI = 0.57–0.98, *p* = 0.03) and gender (OR = 0.71, 95%CI = 0.53–0.95, *p* = 0.02) both moderated the missed appointment rate, such that missed visits were more likely in the intervention group compared to the control group for virtual appointments and men, while less likely for in-person appointments and women. For the cancelation rate in primary care, only modality of appointment was a significant moderator (OR = 1.34, 95%CI = 1.06–1.69, *p* = 0.02), where virtual appointments in the nudge arms were less likely to be canceled than in the control group, and more likely for in-person appointments. In mental health, the only significant moderator was gender for canceled appointment rate (OR = 0.73, 95%CI = 0.53–0.99, *p* = 0.04), where both men and women in the intervention group were less likely to cancel appointments than in the control group, but men more so than women.

For sensitivity analyses, results were generally very similar with two minor exceptions. First, in primary care, when restricting to the patients’ first appointment, the canceled appointment rate was lower for the intervention arms compared to the control arm (OR = 0.88, 95%CI = 0.78–1.00, *p* = 0.04) in the adjusted model. Second, in mental health, when restricting to the patients’ first appointment, the missed appointment rate for arm C (Consequences for Others) was lower compared to that of the control arm (OR = 0.67, 95%CI = 0.45–0.98, *p* = 0.04).

## DISCUSSION

In this yearlong pragmatic trial composed of tens of thousands of VA patients and their appointments in primary care and mental health, we found no effect of incorporating nudges in appointment reminder letters on measures relevant to outpatient appointment attendance, specifically the rate of missed appointments and rate of canceled appointments. The lack of effectiveness of incorporating nudges was seen consistently in comparing all four intervention arms to the control, in comparing each individual intervention arm to the control arm, and in both primary care and mental health, the two largest clinic types in VA. While this was a negative trial, we believe our findings are revealing and important to disseminate, particularly in light of recent analyses suggesting substantial publication bias in the realm of nudge interventions, and perhaps even null effects after accounting for this bias.^42,43^

As a pragmatic trial, our findings may have been impacted by several real-world issues within the VA healthcare system. First, a number of veterans were in more than one study arm, particularly among patients in mental health. Psychiatric comorbidity among VA-using veterans is common, and this likely contributed to patients being seen by multiple mental health providers.^44^ Second, patients’ exposure to the intervention was limited compared to what they would have received in a more traditional efficacy trial. For instance, formatting restrictions in the systems used to create reminder letters prevented us from doing more to highlight messages. Furthermore, our trial was only able to incorporate nudges into appointment reminder *letters*. This left other reminders (text, audio, postcard, and—for virtual appointments that became commonplace during the trial—email) untouched by the intervention. As a consequence, patients may have experienced a diluted effect of the nudge due to varying messages being delivered for the same appointment, and they may also have experienced reminder fatigue,^45^ leading some to effectively ignore reminders altogether.

Our trial was also susceptible to a variety of “on-the-ground” changes in healthcare operations that occurred over the course of the trial. COVID-related impacts included a large-scale switch to virtual appointments, and processes related to this change may have diminished relevance and impact of the trial’s different patterns of outreach to patients. For instance, during the trial it became standard practice for providers to call a patient if they did not log into a virtual appointment, thereby potentially creating a new intervention and obviating a missed appointment. We were also unable to quantify the fidelity of the intervention and cannot guarantee that the offices preparing and sending the appointment reminder letters consistently followed standard procedures.

While our pragmatic trial was large, it was nonetheless limited to a single VA healthcare system among patients who were primarily White, male, and older adults. Findings could differ in other patient populations.

It is also plausible that no intervention focused simply on appointment reminders may be able to create significant change in appointment attendance. Financial incentives—whether framed as a gain or loss—can be used to influence health behaviors, though their cost-effectiveness would need to be considered.^46^ A recent systematic review of missed appointments in diabetes clinic appointments found that qualitative studies suggest psychosocial factors playing a prominent role.^47^ It may be, then, that even in the best of circumstances, nudges embedded into appointment reminders are inadequate to resolve the mixture of emotional, cognitive, and behavioral factors involved in triggering missed appointments. In that case, it may be up to health systems and their researchers to determine whether to deploy and test interventions that are more costly or higher intensity, or both. Perhaps a 10% missed appointment rate in primary care or a 20% missed appointment rate in mental health clinics is as good as can be practically achieved without addressing the larger social and contextual issues underling missed appointments.

In conclusion, in this large pragmatic trial in primary care and mental health clinics, we found appointment reminder letters incorporating brief behavioral nudges did not affect appointment attendance. Attempts to reduce missed appointments or increase cancelation of appointments that are no longer necessary likely require more complex or intensive interventions.

## Supplementary Information

Below is the link to the electronic supplementary material.

Supplementary file1 (DOCX 21 KB)

## Data Availability

A de-identified, anonymized dataset resulting from this study may be shared. Requests for data access should be made in writing to the corresponding author and provide information on the purpose for accessing the data.
