# Supplementary material for: Using Nudges to Reduce Missed Appointments in Primary Care and Mental Health: a Pragmatic Trial
Source: J Gen Intern Med. 2023 Jun 20;38(Suppl 3):894–904. doi: 10.1007/s11606-023-08131-5 (PMC10356735; doi:10.1007/s11606-023-08131-5)
Supplement: Supplementary file 1 — Supplementary file1 (DOCX 21 KB) [file 11606_2023_8131_MOESM1_ESM.docx]

**Predicted Probability for Missed Appointments and Canceled Appointments in Primary Care and Mental Health**

|  |  |  |  |  | Unadjusted Model | | Adjusted Model^†^ | |
| --- | --- | --- | --- | --- | --- | --- | --- | --- |
|  |  | Arm | N* | Count (%) | Predicted Probability  (95% CI) | p-value | Predicted Probability (95% CI) | p-value |
| Primary Care | Missed appointment | All | 46,111 | 5,188 (11.3) |  |  |  |  |
|  |  | Control | 9,721 | 1,159 (11.9) | 11.9 (11.3, 13.6) |  | 10.9 (10.0, 13.7) |  |
|  |  | All nudges | 36,390 | 4,029 (11.1) | 11.0 (10.6, 12.0) | 0.35 | 12.2 (11.2, 15.2) | 0.15 |
|  |  | Control | 9,721 | 1,159 (11.9) | 11.9 (11.3, 13.7) |  | 9.3 (08.4, 11.9) |  |
|  |  | Consequences for self | 12,321 | 1,363 (11.1) | 11.0 (10.5, 12.6) | 0.44 | 10.2 (09.3, 12.9) | 0.30 |
|  |  | Consequences for others | 7,492 | 788 (10.5) | 10.4 (09.7, 12.5) | 0.25 | 10.7 (09.7, 13.8) | 0.13 |
|  |  | Combination of all nudges | 8,889 | 944 (10.6) | 10.6 (09.9, 12.7) | 0.31 | 10.2 (09.2, 13.3) | 0.29 |
|  |  | Social norms | 7,688 | 934 (12.1) | 12.1 (11.1, 15.0) | 0.94 | 10.8 (09.6, 14.5) | 0.18 |
|  |  |  |  |  |  |  |  |  |
|  | Canceled appointment | All | 49,598 | 3,487 ( 7.0) |  |  |  |  |
|  |  | Control | 10,339 | 618 ( 6.0) | 6.0 (5.2, 8.4) |  | 5.7 (5.2, 7.3) |  |
|  |  | All nudges | 39,259 | 2,869 ( 7.3) | 7.3 (7.0, 8.3) | 0.29 | 5.3 (4.8, 6.8) | 0.15 |
|  |  | Control | 10,339 | 618 ( 6.0) | 6.0 (5.3, 8.5) |  | 5.7 (5.2, 7.4) |  |
|  |  | Consequences for self | 13,070 | 749 ( 5.7) | 5.8 (5.1, 7.7) | 0.88 | 5.2 (4.7, 6.9) | 0.25 |
|  |  | Consequences for others | 8,223 | 731 ( 8.9) | 8.9 (8.4, 10.6) | 0.04 | 5.4 (4.9, 7.0) | 0.41 |
|  |  | Combination of all nudges | 9,715 | 826 ( 8.5) | 8.5 (8.1, 9.6) | 0.07 | 5.4 (4.9, 7.1) | 0.41 |
|  |  | Social norms | 8,251 | 563 ( 6.8) | 6.9 (6.3, 8.8) | 0.53 | 5.1 (4.6, 6.6) | 0.11 |
|  |  |  |  |  |  |  |  |  |
| Mental Health | Missed appointment | All | 35,420 | 6,992 (19.7) |  |  |  |  |
|  |  | Control | 6,144 | 1,104 (18.0) | 18.0 (16.5, 22.8) |  | 18.9 (17.1, 24.4) |  |
|  |  | All nudges | 29,276 | 5,888 (20.1) | 20.1 (19.6, 21.6) | 0.38 | 21.8 (20.4, 25.9) | 0.21 |
|  |  | Control | 6,144 | 1,104 (18.0) | 18.1 (16.5, 22.8) |  | 19.2 (17.3, 24.9) |  |
|  |  | Consequences for Self | 5,750 | 1,106 (19.2) | 19.3 (18.0, 23.2) | 0.67 | 21.4 (19.4, 27.6) | 0.46 |
|  |  | Consequences for others | 8,131 | 1,779 (21.9) | 21.9 (20.9, 24.9) | 0.16 | 23.9 (22.2, 29.1) | 0.07 |
|  |  | Combination of all nudges | 6,984 | 1,321 (18.9) | 18.9 (18.1, 21.4) | 0.71 | 21.7 (20.3, 25.8) | 0.31 |
|  |  | Social norms | 8,411 | 1,682 (20.0) | 20.0 (19.2, 22.4) | 0.44 | 21.4 (19.9, 25.7) | 0.37 |
|  |  |  |  |  |  |  |  |  |
|  | Canceled appointment | All | 38,945 | 3,525 ( 9.1) |  |  |  |  |
|  |  | Control | 6,843 | 699 (10.2) | 10.3 (9.5, 12.7) |  | 10.3 (9.3, 13.2) |  |
|  |  | All nudges | 32,102 | 2,826 ( 8.8) | 8.8 (8.5, 9.8) | 0.24 | 8.7 (8.0, 11.0) | 0.14 |
|  |  | Control | 6,843 | 699 (10.2) | 10.3 (09.5, 12.7) |  | 10.2 (9.3, 13.2) |  |
|  |  | Consequences for self | 6,343 | 593 ( 9.3) | 9.4 (8.6, 11.8) | 0.59 | 9.2 (8.2, 11.9) | 0.40 |
|  |  | Consequences for others | 8,841 | 710 ( 8.0) | 8.1 (7.4, 10.2) | 0.15 | 7.8 (6.9, 10.7) | 0.07 |
|  |  | Combination of all nudges | 7,648 | 664 ( 8.7) | 8.7 (8.2, 10.2) | 0.25 | 8.2 (7.4, 10.3) | 0.06 |
|  |  | Social norms | 9,270 | 859 ( 9.3) | 9.3 (8.7, 11.1) | 0.51 | 9.6 (8.7, 12.5) | 0.64 |

* N = number of appointments. For calculation of missed appointments, canceled appointments are not included.

† Adjusted models include covariates for age, gender, race, ethnicity, rurality, VA service connection, depression diagnosis in prior 2 years, PTSD diagnosis in prior 2 years, substance use disorder diagnosis in prior 2 years, Elixhauser comorbidity score, CAN 90 score (PC models only), number of prior visits, type of provider, appointment age, appointment modality (in-person vs. virtual), and appointment location (Portland, Vancouver, other).
